# Impact of Carrier Gas Flow Rate on the Synthesis of Monolayer WSe_2_ via Hydrogen-Assisted Chemical Vapor Deposition

**DOI:** 10.3390/ma17102190

**Published:** 2024-05-07

**Authors:** Xuemin Luo, Yanhui Jiao, Hang Li, Qi Liu, Jinfeng Liu, Mingwei Wang, Yong Liu

**Affiliations:** State Key Laboratory of Advanced Technology for Materials Synthesis and Processing, International School of Materials Science and Engineering (ISMSE), State Wuhan University of Technology, Wuhan 430070, China; luoxuemin1123@163.com (X.L.); j1769877205@163.com (Y.J.); gfhang37@gmail.com (H.L.); liuq@whut.edu.cn (Q.L.); liujinf990528@whut.edu.cn (J.L.); wmw1842591883@163.com (M.W.)

**Keywords:** H_2_, monolayer tungsten selenide, CVD

## Abstract

Transition metal dichalcogenides (TMDs), particularly monolayer TMDs with direct bandgap properties, are key to advancing optoelectronic device technology. WSe_2_ stands out due to its adjustable carrier transport, making it a prime candidate for optoelectronic applications. This study explores monolayer WSe_2_ synthesis via H_2_-assisted CVD, focusing on how carrier gas flow rate affects WSe_2_ quality. A comprehensive characterization of monolayer WSe_2_ was conducted using OM (optical microscope), Raman spectroscopy, PL spectroscopy, AFM, SEM, XPS, HRTEM, and XRD. It was found that H_2_ incorporation and flow rate critically influence WSe_2_’s growth and structural integrity, with low flow rates favoring precursor concentration for product formation and high rates causing disintegration of existing structures. This research accentuates the significance of fine-tuning the carrier gas flow rate for optimizing monolayer WSe_2_ synthesis, offering insights for fabricating monolayer TMDs like WS_2_, MoSe_2_, and MoS_2_, and facilitating their broader integration into optoelectronic devices.

## 1. Introduction

Since the inaugural laboratory synthesis of graphene in 2004 [1], the domain of two-dimensional materials has sparked a widespread exploration frenzy within the global scientific research community, attributed to their extraordinary physicochemical attributes [2,3]. These materials, with their two-dimensional structure just a few atoms thick, demonstrate exceptional surface activity, electronic and optical properties, and noteworthy mechanical stability. With the rapid development of this field, numerous two-dimensional materials such as hexagonal boron nitride (hBN), black phosphorus (also known as “black phosphene”), transition metal dichalcogenides (TMDs), and two-dimensional perovskites have been sequentially unveiled and thoroughly explored [4,5]. They manifest immense potential in diverse application areas including energy conversion, data storage, and optoelectronic device fabrication. In particular, TMDs have emerged as a focal point of scientific inquiry, distinguished by their superior electronic, optical, and mechanical performance levels [6]. Their exhibited tunable bandgap range of 1–2 eV reveals the feasibility of subtle electronic structural modifications via layer number adjustments or the application of external pressures, enabling a shift from indirect to direct bandgaps [7]. For instance, monolayer forms of MoS_2_, MoSe_2_, WS_2_, and WSe_2_ manifest direct bandgaps, conferring advantages in photovoltaic conversion and optical signal processing [8,9], thereby propelling the development of TMDs in applications such as sensor, photodetection, storage devices, and biomedicine [10,11,12,13,14,15].

Mechanical exfoliation techniques for fabricating TMDs are predominantly utilized in the realm of catalysis and foundational studies on the intrinsic attributes of materials [16]. In contrast, chemical vapor deposition (CVD) has been proven to be an effective method for fabricating high-quality TMDs with scalable domain size, controllable thickness, and superior electronic properties [17,18]. Consequently, the fabrication of uniformly distributed and high-quality TMDs has emerged as a focal point in research. Numerous researchers have delved into the pivotal parameters affecting TMDs synthesis via CVD, encompassing variables such as the temperature of growth [19], growth duration, the amount of precursor [20], and the distance between the sources and the growth substrate, achieving noteworthy results [21,22,23].

Notably, recent studies have incrementally uncovered the pivotal influence of hydrogen gas (H_2_) introduction during the CVD process with metal precursors like WO_3_, MoO_3_, and the chalcogens precursors like Se and S, contributing to the crystalline quality enhancement of the resultant TMDs. Zhang et al. employed H_2_-assisted low-pressure chemical vapor deposition (LPCVD) to synthesize WS_2_. This achieved a morphological transition from serrated to straight-edged triangular single-layer WS_2_ sheets, preserving their monocrystalline structure. They posited that H_2_ integration modulates via kinetic effects, facilitating the formation of thermodynamically stable equilateral triangular structures [24]. Subsequent investigations by McCreary et al. into H_2_-assisted growth of WS_2_ revealed that incorporating H_2_ within an Ar atmosphere not only significantly enhanced the photoluminescence intensity, thereby elevating the optical characteristics of WS_2_, but also efficiently minimized the presence of the WO_3_ precursor, which in turn inhibited the oxidative etching observed in monolayer WS_2_ [25]. Sheng et al. investigated the impact of the H_2_/Ar ratio on the growth of large-area WS_2_ films. They revealed that H_2_ in the reaction not only accelerates the process but also mitigates oxidative damage [26]. Ji et al. discovered that H_2_ introduced during growth etches multilayer nuclei on monolayer WS_2_, effectively impeding the genesis of multilayer WS_2_. Furthermore, the study elucidated that defects within the H_2_-WS_2_ grains were suppressed, with H_2_ facilitating the rectification of lattice defects in WS_2_. Due to the energetically unstable nature of these defects, they are readily eradicated by H_2_ during growth, aiding in the WS_2_ lattice reconstitution and thereby enhancing the physical properties of TMDs monolayers grown with H_2_ assistance [27]. These studies conducted a qualitative investigation into the role of hydrogen gas (H_2_) during the growth dynamics of TMDs, underscoring the critical function of hydrogen in the fabrication of high-quality TMDs via CVD method.

WSe_2_ has gathered substantial attention for its unique electronic and optical attributes. It has shown superior photoresponsivity behavior, high carrier mobility, and unique spin–orbit interaction effects [28,29,30]. More significantly, its ambipolar conductivity allows it to be used as either an n-type or p-type semiconductor—considerably broadening its applicability in electronic and optoelectronic devices, signaling its vast potential in the future landscape of nanoelectronics and optoelectronics [31,32]. Compared to other TMDs materials like WS_2_, MoS_2_, and MoSe_2_ [33], research on WSe_2_ growth via hydrogen-assisted CVD is relatively scarce. Given WSe_2_’s significant advantages in performance and potential applications, this study focuses on the growth mechanism of monolayer WSe_2_ under H_2_-assisted CVD, particularly examining the effect of H_2_ flow rate on the morphology of the products on the substrate. A H_2_/Ar gas mixture with 10% H_2_ was used as the carrier gas during the growth phase. An analysis of optical microscopy images of samples obtained under different carrier gas flow rates revealed the crucial impact of flow rate on the growth process. At lower flow rates, the concentration of precursors delivered to the substrate surface dominates product formation, while at higher flow rates, H_2_ causes the decomposition of existing monolayer WSe_2_. Additionally, Raman and photoluminescence spectroscopy, along with AFM results, confirmed the monolayer nature of the synthesized WSe_2_. SEM and XPS analyses provided insights into the elemental composition and valence states, while HRTEM confirmed WSe_2_’s high crystal quality. This research offers a new strategy for precisely controlling H_2_/Ar carrier gas flow rate to grow high-quality monolayer WSe_2_, potentially advancing the practical application of WSe_2_-based optoelectronic devices.

## 2. Materials and Methods

### 2.1. Materials

Tungsten trioxide powder (WO_3_, 99.99% Aladdin, Shanghai, China) and selenium powder (Se, 99%, Aladdin, Shanghai, China) were used as growth precursors. Acetone (AR, Sinopharm Chemical Reagent Co., Ltd., Shanghai, China), anhydrous ethanol (AR, Sinopharm Chemical Reagent Co., Ltd., Shanghai, China) and deionized water were, respectively, utilized for the purification of Si/SiO_2_ substrates (Hefei Kejing Materials Technology Co., Ltd., Hefei, China) on which the materials were synthesized. Argon (Ar gas, 99.999%,Wuhan Newradar Special Gas Co., Ltd., Wuhan, China) was employed as a purging gas and as a protective carrier gas during the temperature ramping stages, while a hydrogen–argon mixture gas (H_2_/Ar, 99.999%, Wuhan Newradar Special Gas Co., Ltd., Wuhan, China) serves as the carrier gas for the growth phase.

### 2.2. The Growth of WSe_2_

Using a single-zone tube furnace, WSe_2_ materials were successfully prepared through chemical vapor deposition (CVD) under atmospheric pressure. Prior to growth, the substrates were ultrasonically cleaned with acetone, ethanol, and deionized water, with each solvent cleaning lasting for 15 min. We weighed out approximately 3600 mg of WO_3_ powder and 820 mg of Se powder. In the experiment, the WO_3_ powder was placed 8 mm from the end of the alumina boat, and the SiO_2_/Si substrate was vertically positioned at the same end of the boat near the WO_3_ side, ensuring the SiO_2_ side faced outward. Additionally, the Se powder was placed in the center of another boat, positioned 16 cm from the heating center at a temperature of 220 °C. After loading the WO_3_ powder and the growth substrates into the boat, we carefully placed it in the center of the tube furnace, ensuring the WO_3_ powder was at the heating center. At room temperature, 300 sccm of Ar gas was injected into the quartz tube, continuing for 30 min. Next, with 100 sccm of Ar carrier gas flowing, the tube furnace temperature was raised to 950 °C within 60 min. Upon reaching 950 °C, we shut off the Ar gas and introduced a 28.3~31.2 sccm H_2_/Ar gas mixed carrier gas containing 10% H_2_, maintaining the temperature for 8 min. Finally, we allowed the tube furnace to naturally cool to below 200 °C, opened the lid, and removed the WSe_2_ samples after cooling to room temperature.

### 2.3. Characterization

Optical microscope (OM) images were obtained using a Sunwoo RX50M microscope (Yuyao, China). Scanning Electron Microscope (SEM) images were captured with a JEM-1400Plus (JEOL, Beijing, China) at an acceleration voltage of 5 kV. Corresponding elemental analysis was performed using a 20 kV acceleration voltage. Photoluminescence (PL) and Raman spectra were measured using a Horiba Raman microscope (Irvine, CA, USA) with a 532 nm laser beam. Atomic Force Microscopy (AFM) image was acquired using a Bruker Dimension FastScan AFM (Billerica, MA, USA) in knockdown mode. X-ray Photoelectron Spectroscopy (XPS) spectra were obtained using a Thermo Scientific Kα XPS spectrometer (Waltham, MA, USA) equipped with a monochromatic Al-Kα X-ray source. X-ray diffraction (XRD) analysis was conducted using a Bruker D8 ADVANCE X-Ray diffractometer (Bruker, Karlsruhe, Germany) with Cu-Kα radiation (λ = 1.54056 Å, 40 kV and 40 mA), with the diffraction angle 2θ range set from 10 to 60°, a scan speed of 0.5 s per step, and a step size of 0.05°. The model used for Transmission Electron Microscopy was a JEOL JEM-1400 Plus electron microscope (JEOL, Beijing, China).

For HRTEM analysis, the WSe_2_ sample was transferred from the growth substrate to the copper grids for electron microscopy using a PMMA-assisted wet transfer technique. Initially, 2 g of NaOH and 50 mL of deionized water were measured to prepare a 1 M NaOH solution. Subsequently, the substrate containing WSe_2_ was placed at the center of a spin coater, 10 μL of PMMA solution was dropped onto it, and it was spin-coated at 2000 rpm for 60 s. The sample was then transferred to a hotplate heated at 85 °C for 15 min to remove residual solvent and to solidify the interface between PMMA and WSe_2_. Afterwards, the sample was immersed in the previously mentioned NaOH solution and wet-etched at 85 °C for 2.5~3 h. During this process, the PMMA/WSe_2_ film floated on the surface of the solution, was subsequently scooped out, and washed several times with deionized water. After drying, the sample was placed on the copper grid, and an appropriate amount of acetone was dropped onto it to remove the PMMA layer, leaving a pure WSe_2_ sample on the copper grid.

## 3. Results and Discussion

**Figure 1:**
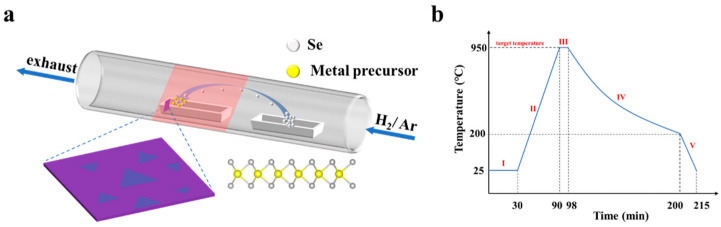
Atmospheric CVD synthesis of WSe_2_. (**a**) Schematic illustration of the single temperature zone tube furnace CVD system used to synthesize of WSe_2_ on the SiO_2_/Si substrate. Illustration of WSe_2_ growing on SiO_2_/Si substrate and cross-sectional atomic structure of monolayer WSe_2_, where yellow and gray spheres represent W and Se atoms, respectively. (**b**) Temperature programming process of heating center.

Figure 1 illustrates the synthesis of WSe_2_ on SiO_2_/Si substrates via a single-zone tube furnace, utilizing selenium (Se) and tungsten trioxide (WO_3_) powders as sources for selenium and tungsten, respectively. The cross-sectional atomic structure of the monolayer WSe_2_ shows the W atom is sandwiched between two Se atoms, forming a Se-W-Se sandwich structure. The Se powder was placed in an alumina boat upstream of the furnace, and its temperature was controlled by adjusting the distance to the heating center. WO_3_ powder was placed in another alumina boat in the heating area, and the thoroughly cleaned 285 nm SiO_2_/Si substrate was positioned vertically to the direction of gas flow, approximately 8 mm from the WO_3_ powder, to ensure uniform reaction on the substrate. The entire reaction was conducted under ambient pressure with an alternating flow of argon and a hydrogen–argon mixture (10% H_2_). This experiment hinged on the high-temperature sublimation of precursors, their transportation to the substrate via carrier gas, and subsequent solid product formation. In the initial phase of our experiments, we extensively explored the impact of growth temperature of WSe_2_, as detailed in Figure S1. During the experiments to investigate the effect of carrier gas flow rate, we maintained a constant growth temperature at 950 °C. The substrate’s outcomes, influenced by the flow rate, ranged from partially reduced WO_3−x_ grains to regular triangular monolayers of WSe_2_ and hydrogen-decomposed monolayer WSe_2_, with an in-depth mechanism and product analysis provided subsequently. As depicted in Figure 1b, the temperature profile within the furnace core throughout the CVD experiment was characterized by a multi-stage process, encompassing pre-purification, ramp-up, growth, cooling, and sample retrieval, detailed across five phases with respective parameters outlined in Table 1.

WO_3_ exhibits good thermal stability, which poses a challenge for its reduction or decomposition by Se, a reductant with limited efficacy, under elevated temperatures. To address this, hydrogen (H_2_) was introduced during the growth phase to promote the reduction of WO_3_ to the more volatile form, WO_3−x_ [34]. This was followed by the transport of gaseous Se and WO_3−x_ to the substrate’s surface via carrier gas, triggering intricate reactions that culminate in the deposition of solid WSe_2_. The synthesis was conducted within a quartz tube of 35 mm diameter, optimizing the homogenous delivery of the carrier and precursor gases to the substrate due to the tube’s constrained diameter. When the heating zone containing WO_3_ reached the growth temperature of WSe_2_ at 950 °C, a suitable regulated H_2_/Ar gas mixture was introduced, quickly filling the entire sealed space, and reducing the WO_3_ powder to volatile WO_3−x_. The WO_3−x_ gas, facilitated by the carrier gas, migrated to the substrate, forming WO_3−x_ nanoparticles—the nucleation sites. Concurrently, the Se powder reached its sublimation point (approximately 220 °C), transforming into vapor and being conveyed to the substrate surface, where it reacted with WO_3−x_ nanoparticles, yielding WSe_2_ samples. The comprehensive reaction for this phase is delineated as follows:

3Se(s) + WO_3_(s) + H_2_(g)→WSe_2_(s) + H_2_O(g) + SeO_2_(g) (1)

Prior investigations have corroborated that the Gibbs free energy of this reaction is negative at the operational temperature, denoting the spontaneity of this substitution reaction [35].

**Figure 2:**
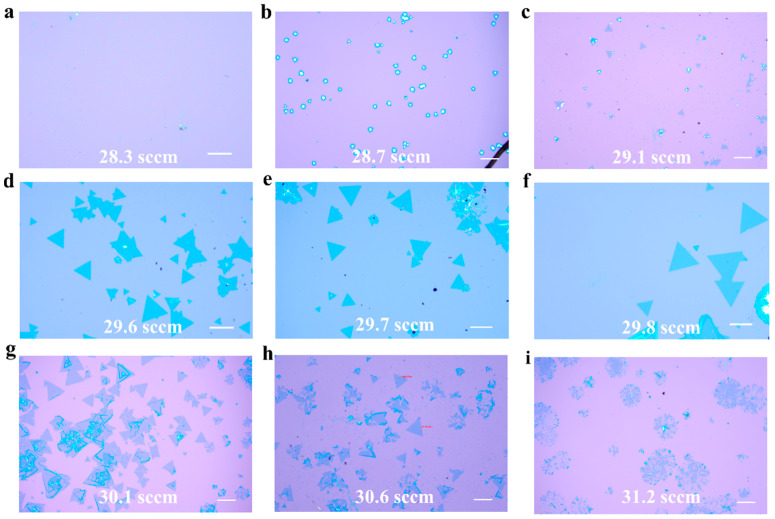
Influence of carrier gas flow rate on products on substrate. The scales of (**a**,**d**) are 100 μm, and the rest are 50 μm.

In our study, a H_2_/Ar mixed gas containing 10% H_2_ was utilized as the carrier gas for the WSe_2_ growth phase. Adjusting the carrier gas’s flow rate yielded WSe_2_ under various conditions depicted in Figure 2. When the flow rate of the carrier gas was too low, the Se vapor could not reach the substrate surface to react, forming the nanoparticles shown in Figure 2a, with a detailed view in Figure S2; the adjustments of the carrier gas flow rate across a wider range are further illustrated in Figure S3. Increasing the flow rate, Figure 2b illustrates the nucleation sites’ periphery turning blue due to limited selenium diffusion to the substrate, producing a small amount of WSe_2_, with WO_3−x_ being the main product. Further increments in flow rate led to complete conversion of some nucleation sites to WSe_2_, depicted by small triangles in Figure 2c, though most of the surface was still covered with particles. In other words, under relatively low carrier gas flow rates, the predominance of Se vapor concentration delivered to the substrate via the H_2_/Ar carrier gas emerged as the critical determinant in steering the reaction dynamics, subsequently affecting the composition of the resultant product. The effects of carrier gas on the transport of Se precursors have also been reported in the existing literature [36].

An optimal increase in flow rate resulted in numerous regular triangular monolayers of WSe_2_, approximately 100 μm in size, as shown in Figure 2d–f, indicating sufficient Se delivery and conversion of most nucleation sites to WSe_2_, albeit with some unreacted sites. Upon optimizing the carrier gas flow rate to a precise range, fine-tuning ceased to impact the synthesis of monolayer WSe_2_ characterized by uniform triangular configurations. This suggests that within this confined parameter range, the production of the targeted samples remains consistent, even with very slight alterations in flow rate. As the flow rate was increased further, as shown in Figure 2g, with a detailed view in Figure S2, although there were still some partially selenized particles on some WSe_2_ flakes, partial decomposition and edge thickening of WSe_2_ monolayers were observed, with extensive decomposition and the loss of sharp triangular edges upon further increases, as seen in Figure 2h. Excessive H_2_ flow as shown in Figure 2i, however, led to significant decomposition of WSe_2_, severely impairing its structural integrity and crystallinity. The results show that excess hydrogen can decompose the monolayer WSe_2_ already present on the substrate.

**Figure 3:**
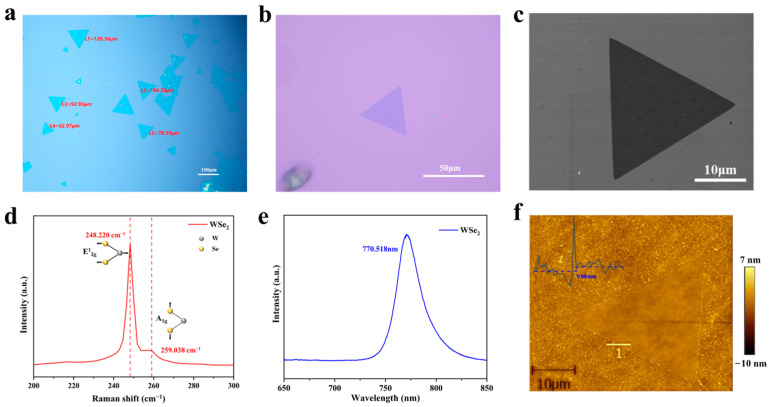
A series of characteristics of monolayer WSe_2_. (**a**) Monolayer WSe_2_ grown on SiO_2_/Si substrates, featuring multiple regular triangular structures. (**b**,**c**) The magnified optical microscopy image and SEM image of an individual WSe_2_, respectively. (**d**) The Raman spectrum of monolayer WSe_2_, with insets at 248.220 cm^−1^ and 259.038 cm^−1^ illustrating the atomic models of in-plane vibrations of W atoms and Se atoms and out-of-plane vibrations of Se atoms in monolayer WSe_2_. (**e**) Shows the photoluminescence spectrum of monolayer WSe_2_. (**f**) The AFM image from a WSe_2_ triangular flake with an inset showing the height distribution along the marked line in the image.

Figure 3a illustrates multiple monolayer WSe_2_ crystals with regular triangular structures grown experimentally on SiO_2_/Si substrates. In this image, each triangle’s lateral dimension exceeds 50 μm and can reach up to 120 μm. Due to the sensitivity of WSe_2_ to the growth substrate, an incompletely cleaned substrate and lattice mismatch between WSe_2_ and SiO_2_/Si substrates could limit the final lateral dimension of the produced WSe_2_. To further confirm the morphology of WSe_2_, we observed a single WSe_2_ at a higher magnification, as shown in Figure 3b. The observed WSe_2_, approximately 45 μm in size, exhibits a regular equilateral triangular structure with atomically sharp edges, indicating good crystallinity. Figure 3c presents an enlarged morphology of a single WSe_2_ obtained using Scanning Electron Microscopy (SEM), with a lateral dimension of about 25 μm. It displays a perfect triangular structure consistent with Figure 3b, with a smooth surface.

It is well known that Raman spectroscopy and photoluminescence (PL) spectroscopy are crucial tools for analyzing TMDs materials, where Raman spectroscopy is primarily used for analyzing atomic vibration modes and doping levels, and it also aids in identifying the layer count of TMDs materials such as WSe_2_, WS_2_, and MoS_2_ [37,38]. Photoluminescence spectroscopy offers information about the bandgap energy of materials, and notably, for monolayer TMDs materials like WSe_2_, WS_2_, and MoS_2_, PL test results are significant indicators of the direct bandgap. To confirm the structure of the WSe_2_ sample, we conducted Raman and photoluminescence tests using a laser with a 532 nm wavelength, as shown in Figure 3d,e. The Raman spectrum displayed two distinct peaks at 248.220 cm^−1^ and 259.038 cm^−1^, corresponding to the in-plane vibrations of W and Se atoms (E^1^_2g_ mode) and the out-of-plane vibrations of Se atoms (A_1g_ mode) in monolayer WSe_2_, respectively [34,38]. The frequency difference between these two peaks is 10.818 cm^−1^. Under illumination with a 532 nm laser, WSe_2_ produced a strong luminescence peak near 770 nm, corresponding to the A exciton absorption of monolayer WSe_2_, indicating its direct bandgap characteristic [29,39,40]. It is noteworthy that bilayer and thicker WSe_2_ usually exhibit an extra indirect bandgap transition peaks at a higher wavelength [34]. Our PL test results showed only a direct bandgap transition peak near 770 nm, with no extra indirect bandgap emission, consistent with recent reports on monolayer WSe_2_. In the PL spectrum of bilayer WSe_2_ shown in Supplementary Figure S4, There is an additional peak at a higher wavelength in addition to the indirect transition peak. Figure 3f shows the Atomic Force Microscopy (AFM) image of WSe_2_, with a thickness of 0.88 nm, which is consistent with the thickness reported in the literature for monolayer WSe_2_ prepared by CVD method. This thickness is slightly greater than that of monolayer WSe_2_ obtained by mechanical exfoliation (0.7 nm) [29,41]. The observed discrepancy in thickness might be attributed to the lattice mismatch between WSe_2_ and the SiO_2_/Si growth substrate, coupled with potential surface states present on the CVD-prepared samples.

**Figure 4:**
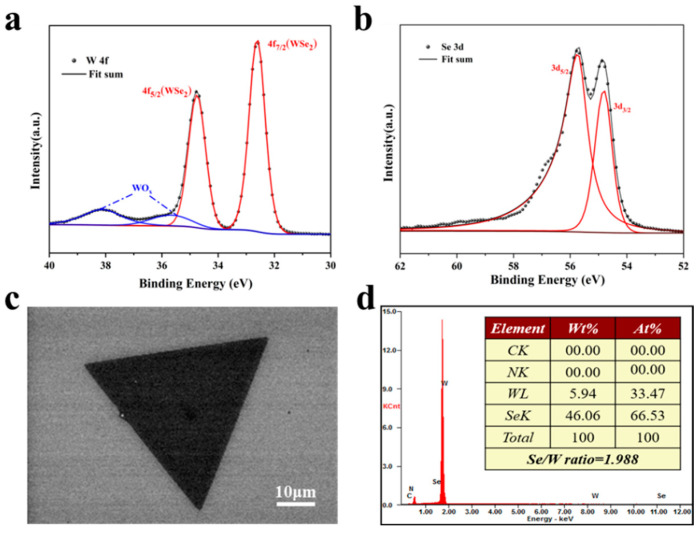
The XPS spectra and SEM-EDS analysis of monolayer WSe_2_. (**a**) The W 4f spectrum reveals two peaks of high intensity at 32.6 eV and 34.8 eV, attributable to the 4f_7/2_ and 4f_5/2_ orbitals of W^4+^ within WSe_2_. (**b**) In the Se 3d spectrum, the binding energies of Se 3d_5/2_ and 3d_3/2_ are located at 55.7 eV and 54.8 eV, respectively. (**c**) The image acquired through SEM-EDS. (**d**) The elemental analysis results of the image in (**c**).

The chemically synthesized WSe_2_ sample was subjected to X-ray Photoelectron Spectroscopy (XPS) for compositional analysis. Figure 4a illustrates the results from the spectral fitting of tungsten in the WSe_2_ samples, revealing two prominent peaks at 32.6 eV and 34.8 eV, assignable to the 4f_7/2_ and 4f_5/2_ orbitals of W^4+^ in WSe_2_ [42]. This spectral fitting evidence a notable transition of the peak from W^6+^ in the WO_3_ precursor to W^4+^, validating the valence change. Additionally, peaks of lesser intensity near 38.2 eV and 35.6 eV were detected, corresponding to the metal oxide WO_x_ [25]. We hypothesize that the presence of trace amounts WO_x_ nanoparticles on the WSe_2_ surface is attributed to the incomplete reduction in the metal oxide precursor WO_3_ during the synthesis of WSe_2_. Figure 4b details the fitting analysis for selenium in WSe_2_, where the binding energies for Se 3d_5/2_ and 3d_3/2_ are identified at 55.68 eV and 54.78 eV, respectively. Supplementary Figure S5 showcases the comprehensive WSe_2_ spectra acquired through XPS testing, featuring four elements: tungsten and selenium from the monolayer WSe_2_, alongside silicon and oxygen from the SiO_2_ substrate [43]. The analytical outcomes from our XPS data on the experimentally derived WSe_2_ align with those of pure phase WSe_2_ reported in the existing literature.

To elucidate the elemental composition of the synthesized WSe_2_ samples, Energy Dispersive Spectroscopy (EDS) analyses were conducted utilizing a SEM at an acceleration voltage of 20 kV, as illustrated in Figure 4c. The data, depicted in the inset of Figure 4d, reveal a Se/W ratio of 1.988 in the WSe_2_ samples, closely aligning with the theoretical stoichiometric ratio of 2:1. This minor deviation from the stoichiometric ratio is ascribed to the presence of selenium vacancies, a prevalent surface defect in WSe_2_ synthesized via CVD, leading to a marginally reduced selenium content.

Significantly, the XPS examination of monolayer WSe_2_ films, stored for an extended period exceeding one month and depicted in Supplementary Figure S6, identified the emergence of two novel peaks at 33.8 eV and 36.0 eV within the tungsten spectrum. Correlation with the literature suggests these peaks are attributed to the transitional states between hexavalent tungsten (W^6+^) in tungsten trioxide (WO_3_) and tetravalent tungsten (W^4+^) in WSe_2_, indicative of the signals produced by partially oxidized WSe_2_ [25]. Subsequently, an annealing intervention was applied to this specimen (situated at the center of heating at 200 °C, subjected to preliminary purification within the tube furnace, and maintained under a 100 sccm flow of argon gas for 300 min). The XPS analysis post-annealing, aligning with the observations in Figure 4a, substantiates that (1) specimens conserved in non-vacuum conditions undergo partial oxidation by environmental oxygen over time, and (2) the annealing process significantly purges impurities, culminating in the amelioration of the crystal quality of the samples. The optical spectra of WSe_2_ before and after annealing are shown in Figure S7, which are consistent with XPS results.

**Figure 5:**
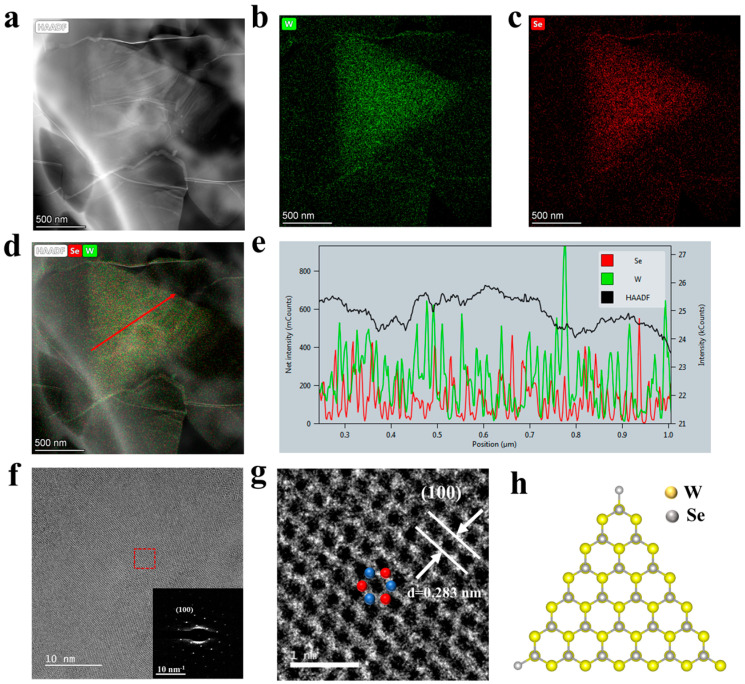
The surface element distribution and microstructural analysis of WSe_2_. (**a**) The HAADF-STEM image of WSe_2_. (**b**,**c**) The distribution of W and Se elements in the area shown in (**a**), respectively. (**d**) An overlay view of the HAADF image with the element distribution. (**e**) The line-scan results of the elements, following the direction of the red arrow marked in (**d**). (**f**) The HRTEM image of a monolayer WSe_2_. The inset shows the SAED pattern along the [001] zone axis, corresponding to the region highlighted by the red dashed box. (**g**) An enlarged view of the area within the red dashed box in (**f**), where the blue spheres represent W atoms and the red ones represent Se atoms. (**h**) A schematic diagram of the planar structure of monolayer WSe_2_.

To investigate the microscopic crystal structure of WSe_2_, the High-Angle Annular Dark-Field Scanning Transmission Electron Microscopy (HAADF-STEM), the High-Resolution Transmission Microscope (HRTEM), and the Selective Area Electron Diffraction (SAED) studies were conducted. In our HAADF-STEM analysis, we concentrated on smaller domains to facilitate a comprehensive examination of the entire domain. The HAADF-STEM image, as shown in Figure 5a, reveals the regular triangular morphology of the WSe_2_ samples. To further elucidate the chemical composition within these domains, we employed EDS for elemental mapping of the triangular domains. These mapping images indicate a nearly uniform distribution of W and Se elements throughout the triangular domains (as seen in Figure 5b,c). The corresponding surface scans (Figure 5b–d) and line scan (Figure 5e) indicate a uniform distribution of W and Se elements on the sample surface, confirming the high quality of the prepared WSe_2_ sample. Supplementary Figure S8 presents the corresponding EDS elemental spectra, which show good compatibility with WSe_2_.

HRTEM was used to further analyze the microstructure of WSe_2_, as shown in Figure 5f,g. A detailed element distribution spectrum is shown in Supplementary Information Figure S8. In Figure 5f, the SAED test on the region outlined by the red dashed box reveals the typical SAED pattern of 2H-WSe_2_ crystals (as illustrated in the inset of Figure 5f). The hexagonal symmetry of the diffraction spots from the (100) plane corresponds to the hexagonal symmetry of the WSe_2_ lattice structure in the [001] zone axis, confirming the single-crystalline nature of the WSe_2_ sample with a hexagonal lattice structure. In Figure 5g, the magnified view within the red dashed box, when correlated with the previous diffraction peak analysis, reveals that the interplanar spacing of the (100) plane is 0.283 nm [44]. This measurement consistent with the standard values for WSe_2_. The X-ray diffraction (XRD) analysis of the monolayer WSe_2_, presented in Supplementary Figure S9, identified the (002), (006), and (008) crystallographic planes, indicative of its layered structure [35,45]. This observation is in alignment with findings obtained through HRTEM. Additionally, Figure 5g clearly depicts the six-membered ring structure, aligning with the inherent hexagonal lattice structure of 2H-phase WSe_2_ (as shown in Figure 5h), where W and Se atoms are alternately arranged to form hexagonal rings [46].

## 4. Conclusions

In this study, we successfully synthesized monolayer WSe_2_ with a regular triangular morphology using a H_2_-assisted CVD technique. The experiment utilized a H_2_/Ar mixed gas, with the H_2_ ratio fixed at 10%, as the carrier gas during the growth phase. Our findings indicate that slight adjustments to the carrier gas flow rate during the growth stage significantly impact the composition of the products formed on the substrate. Specifically, at lower carrier gas flow rates, the concentration of Se vapor transported to the substrate by the H_2_/Ar carrier gas became the dominant factor driving the reaction, thereby influencing the final product composition. As the carrier gas flow rate increased, the concentration of Se vapor involved in the reaction rose, enhancing the selenization of the WO_3−x_ nucleation sites. When the carrier gas flow rate was optimized within a specific range, minor adjustments did not affect the achievement of monolayer WSe_2_ with a regular triangular morphology, indicating that a stable preparation of the desired sample could be maintained with minor flow rate adjustments within this narrow window. However, further increasing the H_2_/Ar carrier gas flow rate intensified the impact of H_2_ in the carrier gas on the final product, particularly in decomposing the already formed monolayer WSe_2_, a phenomenon that became more pronounced with increased carrier gas flow rates. This study not only provides significant insights for the preparation of monolayer WSe_2_ via H_2_-assisted CVD but also serves as a reference for the fabrication of other monolayer TMDs materials such as WS_2_, MoS_2_, and MoSe_2_, laying the groundwork for the future application of these materials in optoelectronic devices.

## Figures and Tables

**Table 1:** Parameters for each stage in the synthesis of WSe_2_ via multi-step H_2_-assisted CVD.

Time (min)	Stage	Duration (min)	Ar (sccm)	H_2_/Ar (sccm)	Temperature (°C)
0–30	Ⅰ	30	300	0	25–25
30–90	Ⅱ	60	100	0	25–950
90–98	Ⅲ	8	0	28.3~31.2	950–950
98–200	Ⅳ	102	100	0	950–200
200–215	Ⅴ	15	0	0	200–25

## Data Availability

Data are available in a publicly accessible repository that does not issue DOIs or upon request from the corresponding author.

## Supplementary Materials

The following supporting information can be downloaded at: https://www.mdpi.com/article/10.3390/ma17102190/s1, Table S1: TMDs materials were prepared by CVD method; Figure S1: The effect of growth temperature on WSe_2_ growth. (a) 900 °C, (b) 925 °C, (c) 950 °C and (d) 975 °C; Figure S2: The optical microscope images at higher magnifications related to Figure 2; Figure S3: Supplement to the effect of carrier gas flow rate on products on substrate.(a) 27.6 sccm, (b) 27.9 sccm, (c) 28.3 sccm, (d) 31.2 sccm, (e) 31.6 sccm and (f) 31.9 sccm; Figure S4: PL spectrum of WSe_2_. (a) bilayer WSe_2_, (b) thickness-dependent PL spectra of WSe_2_; Figure S5: The comprehensive WSe_2_ spectrum acquired through XPS testing; Figure S6: The XPS fitting analysis of W element in WSe_2_ films, stored under non-vacuum conditions for an extended period exceeding one month; Figure S7: WSe_2_ Optical spectra of samples before and after annealing. (a) the photoluminescence spectra of samples, (b) and (c) the Raman spectra of samples; Figure S8: WSe_2_ elemental distribution spectrum conducted on the sample corresponding to Figure 5a under HAADF-STEM mode; Figure S9: The XRD analysis of monolayer WSe_2_. References [20,25,26,32,34,35,43,45,47,48,49,50,51] are cited in the Supplementary Materials.

## References

[1] Novoselov K.S., Geim A.K., Morozov S.V., Jiang D., Zhang Y., Dubonos S.V., Grigorieva I.V., Firsov A.A. (2004). Electric Field Effect in Atomically Thin Carbon Films. Science.

[2] Gupta A., Sakthivel T., Seal S. (2015). Recent development in 2D materials beyond graphene. Prog. Mater. Sci..

[3] Shanmugam V., Mensah R.A., Babu K., Gawusu S., Chanda A., Tu Y., Neisiany R.E., Försth M., Sas G., Das O. (2022). A Review of the Synthesis, Properties, and Applications of 2D Materials. Part. Part. Syst. Charact..

[4] Thakur N., Kumar P., Kumar S., Singh A.K., Sharma H., Thakur N., Dahshan A., Sharma P. (2024). A review of two-dimensional inorganic materials: Types, properties, and their optoelectronic applications. Prog. Solid State Chem..

[5] Castellanos-Gomez A., Duan X.F., Fei Z., Gutierrez H.R., Huang Y., Huang X.Y., Quereda J., Qian Q., Sutter E., Sutter P. (2022). Van der Waals heterostructures. Nat. Rev. Methods Primers.

[6] Pham P.V., Bodepudi S.C., Shehzad K., Liu Y., Xu Y., Yu B., Duan X.F. (2022). 2D Heterostructures for Ubiquitous Electronics and Optoelectronics: Principles, Opportunities, and Challenges. Chem. Rev..

[7] Desai S.B., Seol G., Kang J.S., Fang H., Battaglia C., Kapadia R., Ager J.W., Guo J., Javey A. (2014). Strain-induced indirect to direct bandgap transition in multilayer WSe_2_. Nano Lett..

[8] Thakar K., Lodha S. (2020). Optoelectronic and photonic devices based on transition metal dichalcogenides. Mater. Res. Express.

[9] Liang S.J., Cheng B., Cui X.Y., Miao F. (2020). Van der Waals Heterostructures for High-Performance Device Applications: Challenges and Opportunities. Adv. Mater..

[10] Mandyam S.V., Kim H.M., Drndić M. (2020). Large area few-layer TMD film growths and their applications. J. Phys. Mater..

[11] Maniyar A., Choudhary S. (2020). Visible region absorption in TMDs/phosphorene heterostructures for use in solar energy conversion applications. RSC Adv..

[12] Kim T., Kang D., Lee Y., Hong S., Shin H.G., Bae H., Yi Y., Kim K., Im S. (2020). 2D TMD Channel Transistors with ZnO Nanowire Gate for Extended Nonvolatile Memory Applications. Adv. Funct. Mater..

[13] Chen H.Y., Wan T.Q., Zhou Y., Yan J.M., Chen C.S., Xu Z.H., Zhang S.G., Zhu Y., Yu H.Y., Chai Y. (2023). Highly Nonlinear Memory Selectors with Ultrathin MoS_2_/WSe_2_/MoS_2_ Heterojunction. Adv. Funct. Mater..

[14] Wang J.Y., Ilyas N., Ren Y.J., Ji Y., Li S., Li C.C., Liu F.C., Gu D., Ang K.W. (2024). Technology and Integration Roadmap for Optoelectronic Memristor. Adv. Mater..

[15] Karunakaran S., Pandit S., Basu B., De M. (2018). Simultaneous Exfoliation and Functionalization of 2H-MoS_2_ by Thiolated Surfactants: Applications in Enhanced Antibacterial Activity. J. Am. Chem. Soc..

[16] Kashyap P.K., Kumar A., Srivastava R., Gupta S., Gupta B.K. (2021). A Facile Liquid Phase Exfoliation of Tungsten Diselenide using Dimethyl Sulfoxide as Polar Aprotic Solvent to Produce High-quality Nanosheets. ChemNanoMat.

[17] Samaniego-Benitez J.E., Mendoza-Cruz R., Bazán-Díaz L., Garcia-Garcia A., Arellano-Jimenez M.J., Perez-Robles J.F., Plascencia-Villa G., Velázquez-Salazar J.J., Ortega E., Favela-Camacho S.E. (2020). Synthesis and structural characterization of MoS_2_ micro pyramids. J. Mater. Sci..

[18] Ko H., Kim H.S., Ramzan M.S., Byeon S., Choi S.H., Kim K.K., Kim Y.H., Kim S.M. (2019). Atomistic mechanisms of seeding promoter-controlled growth of molybdenum disulphide. 2D Mater..

[19] Suzuki H., Hashimoto R., Misawa M., Liu Y.J., Kishibuchi M., Ishimura K., Tsuruta K., Miyata Y., Hayashi Y. (2022). Surface Diffusion-Limited Growth of Large and High-Quality Monolayer Transition Metal Dichalcogenides in Confined Space of Microreactor. ACS Nano.

[20] Zafar A., Zafar Z., Zhao W.W., Jiang J., Zhang Y., Chen Y.F., Lu J.P., Ni Z.H. (2019). Sulfur-Mastery: Precise Synthesis of 2D Transition Metal Dichalcogenides. Adv. Funct. Mater..

[21] Sierra J.F., Fabian J., Kawakami R.K., Roche S., Valenzuela S.O. (2021). Van der Waals heterostructures for spintronics and opto-spintronics. Nat. Nanotechnol..

[22] Cain J.D., Shi F., Wu J., Dravid V.P. (2016). Growth Mechanism of Transition Metal Dichalcogenide Monolayers: The Role of Self-Seeding Fullerene Nuclei. ACS Nano.

[23] Shim G.W., Hong W., Yang S.Y., Choi S.Y. (2017). Tuning the catalytic functionality of transition metal dichalcogenides grown by chemical vapour deposition. J. Mater. Chem. A.

[24] Zhang Y., Zhang Y.F., Ji Q.Q., Ju J., Yuan H.T., Shi J.P., Gao T., Ma D.L., Liu M.X., Chen Y.B. (2013). Controlled Growth of High-Quality Monolayer WS_2_ Layers on Sapphire and Imaging Its Grain Boundary. ACS Nano.

[25] McCreary K.M., Hanbicki A.T., Jernigan G.G., Culbertson J.C., Jonker B.T. (2016). Synthesis of Large-Area WS_2_ monolayers with Exceptional Photoluminescence. Sci. Rep..

[26] Sheng Y.W., Tan H.J., Wang X.C., Warner J.H. (2017). Hydrogen Addition for Centimeter-Sized Monolayer Tungsten Disulfide Continuous Films by Ambient Pressure Chemical Vapor Deposition. Chem. Mater..

[27] Ji H.G., Lin Y.C., Nagashio K., Maruyama M., Solís-Fernandez P., Aji A.S., Panchal V., Okada S., Suenaga K., Ago H. (2018). Hydrogen-Assisted Epitaxial Growth of Monolayer Tungsten Disulfide and Seamless Grain Stitching. Chem. Mater..

[28] Pradhan N.R., Rhodes D., Memaran S., Poumirol J.M., Smirnov D., Talapatra S., Feng S., Perea-Lopez N., Elias A.L., Terrones M. (2015). Hall and field-effect mobilities in few layered p-WSe_2_ field-effect transistors. Sci. Rep..

[29] Buchkov K., Rafailov P., Minev N., Videva V., Strijkova V., Lukanov T., Dimitrov D., Marinova V. (2024). Metatungstate Chemical Vapor Deposition of WSe_2_: Substrate Effects, Shapes, and Morphologies. Crystals.

[30] Regan E.C., Wang D., Paik E.Y., Zeng Y., Zhang L., Zhu J., MacDonald A.H., Deng H., Wang F. (2022). Emerging exciton physics in transition metal dichalcogenide heterobilayers. Nat. Rev. Mater..

[31] Jadczak J., Kutrowska J., Schindler J.J., Debus J., Watanabe K., Taniguchi T., Ho C.H., Bryja L. (2021). Investigations of Electron-Electron and Interlayer Electron-Phonon Coupling in van der Waals hBN/WSe_2_/hBN Heterostructures by Photoluminescence Excitation Experiments. Materials.

[32] Alahmadi M., Mahvash F., Szkopek T., Siaj M. (2021). A two-step chemical vapor deposition process for the growth of continuous vertical heterostructure WSe_2_/h-BN and its optical properties. RSC Adv..

[33] Hwang Y., Shin N. (2019). Hydrogen-assisted step-edge nucleation of MoSe_2_ monolayers on sapphire substrates. Nanoscale.

[34] Huang J.K., Pu J., Hsu C.L., Chiu M.H., Juang Z.Y., Chang Y.H., Chang W.H., Iwasa Y., Takenobu T.S., Li L.J. (2014). Large-Area Synthesis of Highly Crystalline WSe_2_ Monolayers and Device Applications. ACS Nano.

[35] Kim H., Yun S.J., Park J.C., Park M.H., Park J.H., Kim K.K., Lee Y.H. (2015). Seed Growth of Tungsten Diselenide Nanotubes from Tungsten Oxides. Small.

[36] Gong Y.J., Ye G.L., Lei S.D., Shi G., He Y.M., Lin J.H., Zhang X., Vajtai R., Pantelides S.T., Zhou W. (2016). Synthesis of Millimeter-Scale Transition Metal Dichalcogenides Single Crystals. Adv. Funct. Mater..

[37] Iqbal M.W., Shahzad K., Akbar R., Hussain G. (2020). A review on Raman finger prints of doping and strain effect in TMDCs. Microelectron. Eng..

[38] Terrones H., Corro E.D., Feng S., Poumirol J.M., Rhodes D., Smirnov D., Pradhan N.R., Lin Z., Nguyen M.A.T., Elías A.L. (2014). New First Order Raman-active Modes in Few Layered Transition Metal Dichalcogenides. Sci. Rep..

[39] Xu Z.Q., Zhang Y.P., Wang Z.Y., Shen Y.T., Huang W.C., Xia X., Yu W.Z., Xue Y.Z., Sun L.T., Zheng C.X. (2016). Atomically thin lateral p–n junction photodetector with large effective detection area. 2D Mater..

[40] Tonndorf P., Schmidt R., Böttger P., Zhang X., Börner J., Liebig A., Albrecht M., Kloc C., Gordan O., Zahn D.R.T. (2013). Photoluminescence emission and Raman response of monolayer MoS_2_, MoSe_2_, and WSe_2_. Opt. Express.

[41] Fang H., Chuang S., Chang T.C., Takei K., Takahashi T., Javey A. (2012). High-Performance Single Layered WSe_2_ p-FETs with Chemically Doped Contacts. Nano Lett..

[42] Boscher N.D., Carmalt C.J., Parkin I.P. (2006). Atmospheric pressure chemical vapor deposition of WSe_2_ thin films on glass—Highly hydrophobic sticky surfaces. J. Mater. Chem..

[43] Wang X.L., Gong Y.J., Shi G., Chow W.L., Keyshar K., Ye G., Vajtai R., Lou J., Liu Z., Ringe E. (2014). Chemical Vapor Deposition Growth of Crystalline Monolayer MoSe_2_. ACS Nano.

[44] Zhang Z.W., Chen P., Duan X.D., Zang K.T., Luo J., Duan X.F. (2017). Robust epitaxial growth of two-dimensional heterostructures, multi heterostructures, and superlattices. Science.

[45] Zhang Y.X., Wang Y.H., Xiong Z.Z., Zhang H.J., Liang F. (2018). Preparation and characterization of WSe_2_ nano-films by magnetron sputtering and vacuum selenization. Nanotechnology.

[46] Yang T., Zheng B., Wang Z., Xu T., Pan C., Zou J., Zhang X., Qi Z., Liu H., Feng Y. (2017). Van der Waals epitaxial growth and optoelectronics of large-scale WSe_2_/SnS_2_ vertical bilayer p–n junctions. Nat. Commun..

[47] Lee Y.H., Zhang X.Q., Zhang W.J., Chang M.T., Lin C.T., Chang K.D., Yu Y.C., Wang J.T.W., Chang C.S., Li L.J. (2012). Synthesis of Large-Area MoS_2_ Atomic Layers with Chemical Vapor Deposition. Adv. Mater..

[48] Shaw C., Zhou H., Chen Y., Weiss N.O., Liu Y., Huang Y. (2014). Chemical vapor deposition growth of monolayer MoSe_2_ nanosheets. Nano Res..

[49] Liu B.L., Fathi M., Chen L., Abbas A., Ma Y.Q., Zhou C.W. (2015). Chemical Vapor Deposition Growth of Monolayer WSe_2_ with Tunable Device Characteristics and Growth Mechanism Study. ACS Nano.

[50] Qiao P., Xia J., Li X., Li Y., Cao J., Zhang Z., Lu H., Meng Q., Li J., Meng X.M. (2023). Epitaxial van der Waals contacts of 2D TaSe_2_-WSe_2_ metal-semiconductor heterostructures. Nanoscale.

[51] Wang X., Li Y., Zhuo L., Zheng J., Peng X., Jiao Z., Xiong X., Han J., Xiao W. (2018). Controllable growth of two-dimensional WSe_2_ using salt as co-solvent. CrystEngComm..

