# Supplementary material for: Impact of Carrier Gas Flow Rate on the Synthesis of Monolayer WSe2 via Hydrogen-Assisted Chemical Vapor Deposition
Source: Materials (Basel). 2024 May 7;17(10):2190. doi: 10.3390/ma17102190 (PMC11123087; doi:10.3390/ma17102190)
Supplement: Supplementary file 1 [file materials-17-02190-s001.zip › materials-2936046-supplementary.pdf]

Supporting Information

Impact of Carrier Gas Flow Rate on the Synthesis of Monolayer WSe_2 via Hydrogen-Assisted Chemical Vapor Deposition

Xuemin Luo, Yanhui Jiao, Hang Li, Qi Liu, Jinfeng Liu, and Mingwei Wang and Yong Liu *

International School of Materials Science and Engineering (ISMSE), State Key Laboratory of Advanced Technology for Materials Synthesis and Processing, Wuhan University of Technology, Wuhan 430070, China;
luoxuemin1123@163.com (X.L.); j1769877205@163.com (Y.J.);
gfhang37@gmail.com (H.L.); liuq@whut.edu.cn (Q.L.);
liujinf990528@whut.edu.cn (J.L.); wmw1842591883@163.com (M.W.).
*Correspondence: liuyong3873@whut.edu.cn

Table S1 TMDs materials were prepared by CVD method

**Materials**	Precursors	Temperature(℃)	Carrier gas type	References
MoS_2	MoO_3, S powder	650	Ar	Ref.[1]
WS_2	WO_3, S powder	850	Ar/H_2(24-12.5:1)	Ref.[2]
$MoSe_2$	MoO_3, Se powder	750	Ar/H_2(13:1)	Ref.[3]
$MoSe_2$	MoO_3, Se powder	750	Ar/H_2(17:3)	Ref.[4]
WS_2	WO_3, S powder	1100	Ar	Ref.[5]
WSe_2	WO_3, Se powder	925	Ar/H_2(8:2)	Ref.[6]
WSe_2	WO_3, Se powder	950	Ar/H_2(16:1)	Ref.[7]
WSe_2	WO_3, Se powder	950	Ar/H_2(64:3)	Ref.[8]
WSe_2	WO_3, Se powder	950	Ar/H_2(19:1)	Ref.[9]
WSe_2	WO_3, NaCl, Se	850	Ar/H_2(9:1)	Ref.[10]

Comparing the parameters used in the preparation of WS_2, $MoSe_2$, MoS_2, and $MoSe_2$ materials via the Chemical Vapor Deposition (CVD) method in existing reports, it can be observed that they all employ metal oxide precursors (WO_3 or MoO_3 powders) and chalcogen elements (S and Se powders) as precursors. Under high temperatures, the precursor vapors, after being sublimated, are transported to the substrate surface through inert gases or a mix containing a small amount of reductive gas H_2. This leads to the reaction on the substrate surface, ultimately yielding the desired products.

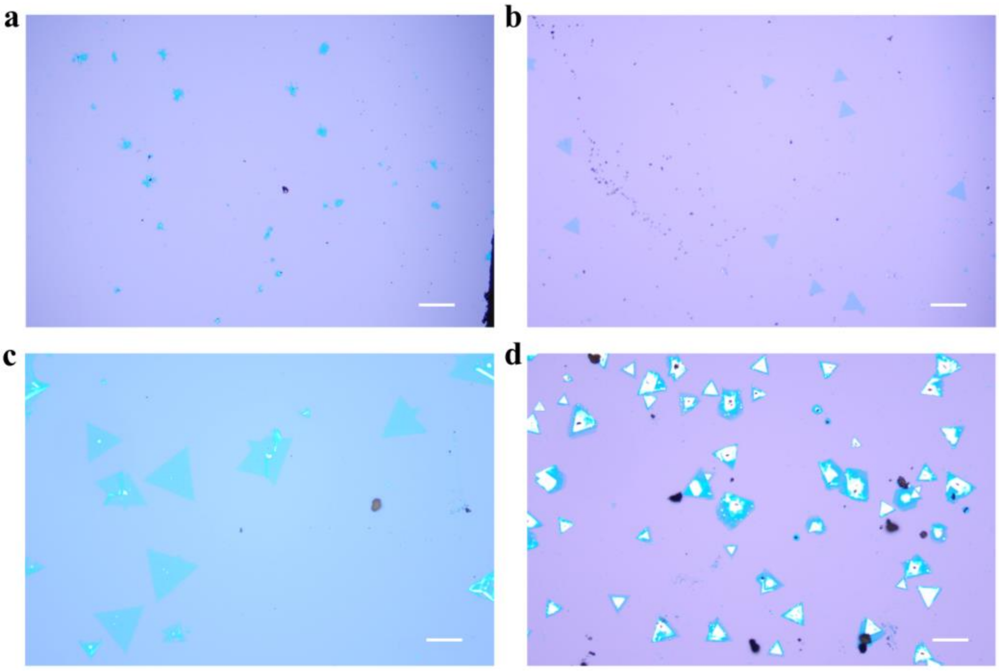

Figure S1. The effect of growth temperature on WSe_2 growth. (a) 900°C, (b) 925°C, (c) 950°C and (d) 975°C, the scales are 50 μm.

In the initial exploratory phase of our experiments, we have already investigated the optimal temperature for the growth of WSe_2, under a constant carrier gas flow rate of 29.7 sccm. Optical microscope images of representative WSe_2 synthesized at 900°C, 925°C, 950°C, and 975°C are displayed in **Figure S1**a-d respectively. As shown in **Figure S1**a, at 900°C, only amorphous samples were obtained on the substrate. Upon increasing the temperature to 925°C, as shown in **Figure S1**b, small triangular formations began to appear on the substrate. Further elevation of the temperature to 950°C resulted in larger monolayer WSe_2 samples, as shown in **Figure S1**c. At a growth temperature of 975°C, as shown in **Figure S1**d, the substrate was covered with dense, small-sized triangular formations. Following these explorations, we selected 950°C as the growth temperature for WSe_2 in subsequent experiments to investigate the effects of carrier gas flow rate on its growth.

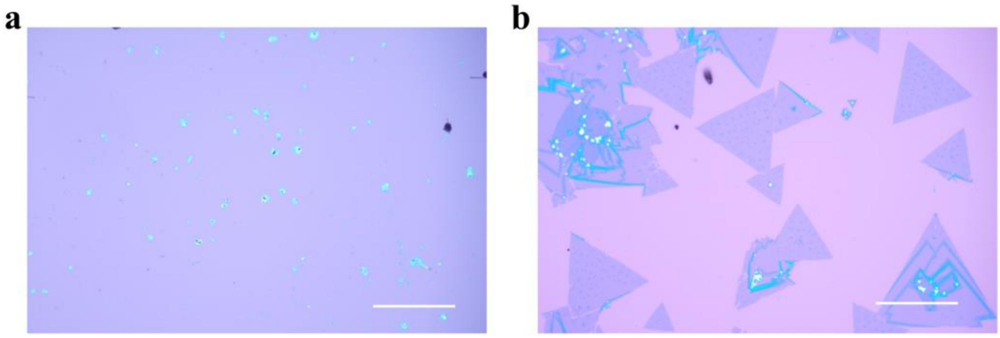

Figure S2. The optical microscope images at higher magnifications related to Figure 2. (**a**) and (**b**) correspond to the optical microscope at higher multiples corresponding to the 2a and 2g samples in Figure 2, respectively. The scales bar are 100 μm.

Figure S2a illustrates the magnified view of the products formed on the substrate under a too low carrier gas flow rate of 28.3 sccm. At this flow rate, the substrate predominantly features

volatile WO_{3-x}, which has been partially reduced by H_2 and then conveyed to the nucleation sites on the substrate via carrier gas. Figure S2b also depicts the scenario under a carrier gas flow rate of 30.1 sccm, slightly above the ideal rate. In this condition, it is evident that a fraction of the monolayer WSe_2 has commenced decomposing, with certain monolayer WSe_2 entities exhibiting thicker layers at their peripheries.

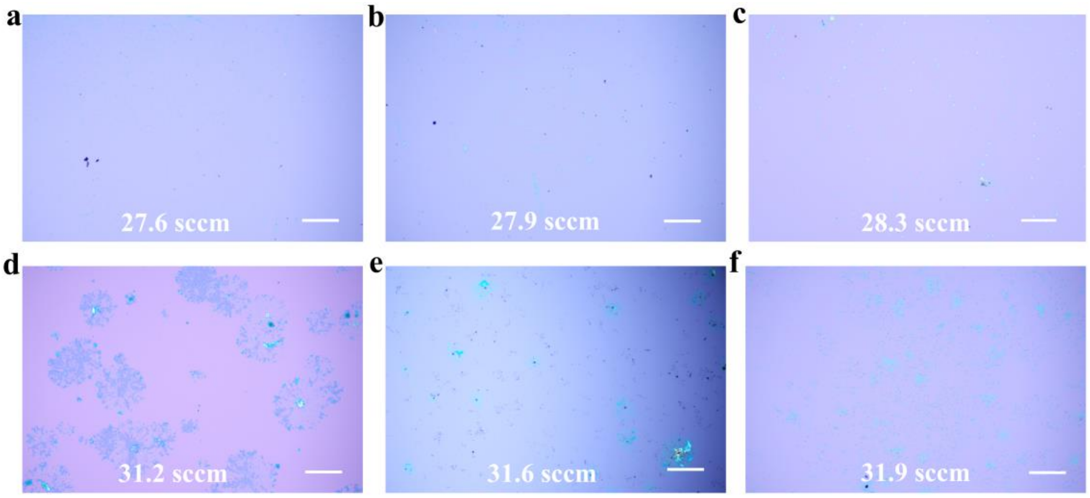

Figure S3. Supplement to the effect of carrier gas flow rate on products on substrate. (a) 27.6 sccm, (b) 27.9 sccm, (c) 28.3 sccm, (d) 31.2 sccm, (e) 31.6 sccm and (f) 31.9 sccm, the scales are 50 μm.

As shown in the **Figure S3**, at low carrier gas flow rates, exemplified in **Figure S3a**, there was essentially no product formation on the substrate. With a slight increase in flow rate, a few nucleation points began to appear. It wasn't until the flow rate reached 28.3 sccm that significant nucleation points were observed on the substrate, establishing 28.3 sccm as the minimum value for fine-tuning the carrier gas flow. Experiments indicated that at excessively high flow rates, such as those above the fine-tuned maximum of 31.2 sccm depicted in **Figure S3e**, the monolayer WSe_2 samples almost entirely decomposed, leaving only a few thicker WSe_2 specimens on the substrate. Further increase in the flow rate led to additional decomposition of the multilayer samples. Thus, we meticulously adjusted the carrier gas within the narrow range of 28.3-31.2 sccm to investigate the effect of carrier gas flow rate on the growth of WSe_2 samples.

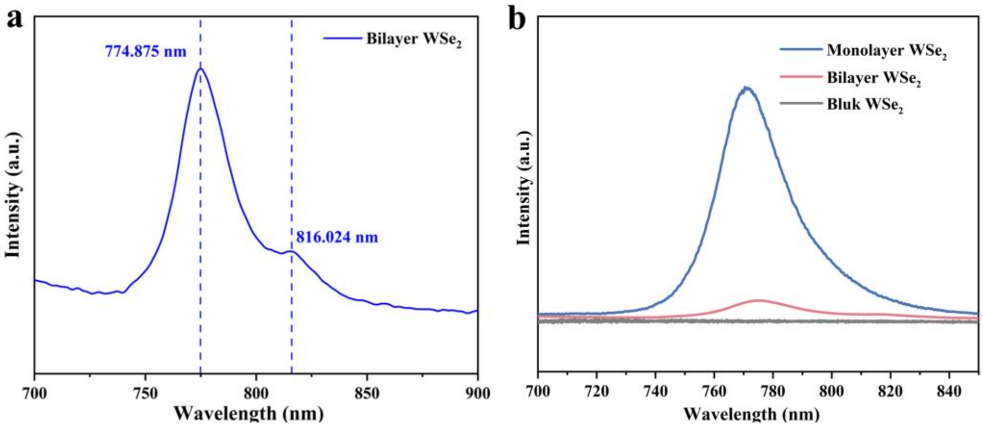

Figure S4. PL spectrum of WSe_2 (a) bilayer WSe_2, (b) thickness-dependent PL spectra of WSe_2.

In addition to the direct transition peak, an additional peak appears at higher wavelengths, corresponding to an indirect transition of the two-layer WSe_2[6]. Furthermore, we conducted PL testing on samples of varying thicknesses. We observed that the intensity of the photoluminescence varied significantly with thickness. Notably, the PL intensity of monolayer samples was significantly higher compared to that of samples with other thicknesses. This finding highlights the distinct optical properties of monolayer TMDs, underscoring their potential for applications where high PL efficiency is crucial.

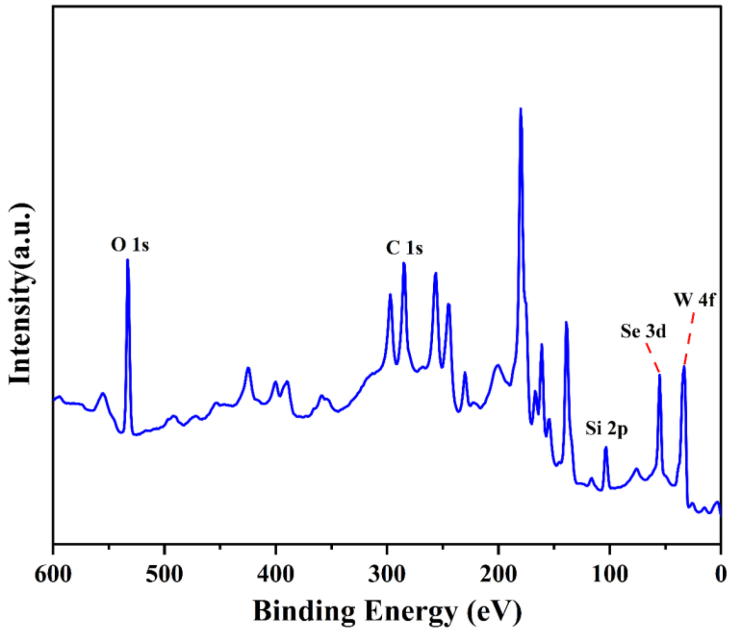

Figure S5. The comprehensive WSe_2 spectrum acquired through XPS testing, featuring four elements: tungsten and selenium from the monolayer WSe_2, alongside silicon and oxygen from the SiO_2 substrate[4].

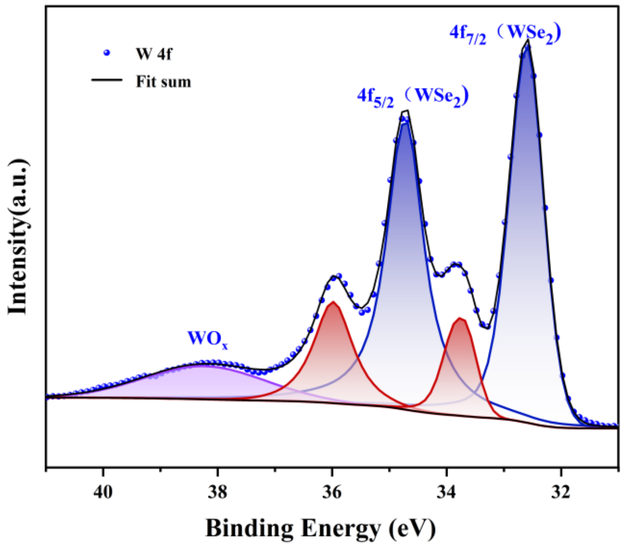

Figure S6. The XPS fitting analysis of W element in WSe_2 films, stored under non-vacuum conditions for an extended period exceeding one month.

Notably, the emergence of two novel peaks at 33.8 eV and 36.0 eV within the tungsten spectrum. Literature correlation suggests these peaks are attributed to the intermediate states transitioning from hexavalent tungsten (W^{6+}) in tungsten trioxide (WO_3) to tetravalent tungsten (W^{4+}) in WSe_2, indicative of the signals produced by partially oxidized WSe_2[11].

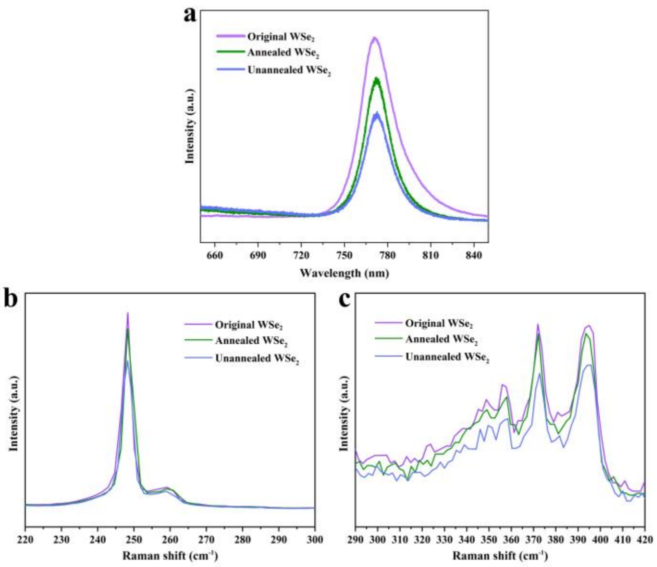

Figure S7 WSe_2 Optical spectra of samples before and after annealing. (a) the photoluminescence spectra of samples, (b) and (c) the Raman spectra of samples.

As shown in Figure S7, Figure S7a displays the photoluminescence (PL) spectra of the sample before and after annealing, while Figure S7b and Figure S7c correspond to the Raman spectra. Figure **S7**c demonstrates that no significant peak appears near 307cm^{-1}, which correlates well with the monolayer nature of the sample. This observation is consistent with findings reported in the literature[6]. It is clearly observable from the spectral data that the sample initially exhibited the highest spectral intensity. However, after being stored for over a month, the spectral intensity (including both PL and Raman spectra) significantly diminished. Furthermore, after the annealing process, the spectral intensity of the sample was moderately enhanced, yet it did not return to its original level. This indicates that the optical properties of the sample evolve over time and are influenced by thermal treatment.

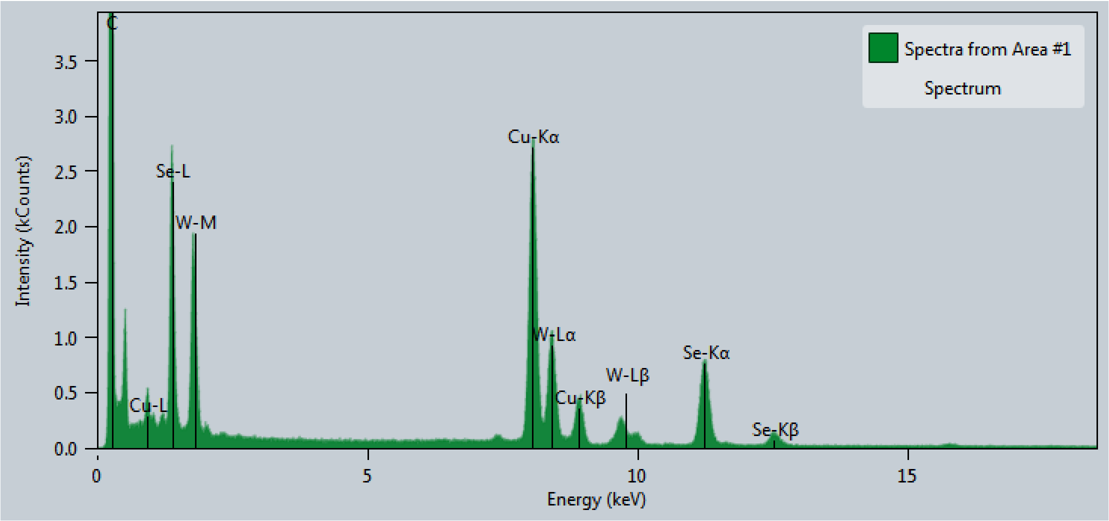

Figure S8. WSe_2 elemental distribution spectrum conducted on the sample corresponding to Figure 5a under HAADF-STEM mode.

EDS analysis was performed on the WSe_2 sample corresponding to Figure 5a utilizing the HAADF-STEM mode. The pronounced carbon (C) peak is attributed to the organic carbon coating on the copper grid, which serves to safeguard the specimen by mitigating the deleterious effects of electron beam exposure. The presence of a copper (Cu) peak is indicative of the copper grid substrate. The spectrum distinctly delineates peaks associated with selenium (Se), including Se-L, Se-Kα, Se-Kβ, and tungsten (W), encompassing W-M, W-Lα, W-L.

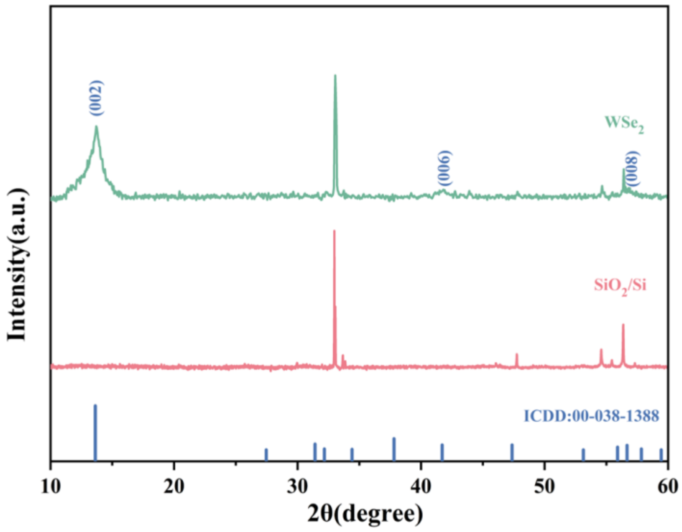

Figure S9. The XRD analysis of monolayer WSe_2.

To gain deeper insights into the structural characteristics of the sample, we conducted X-ray diffraction (XRD) analysis on a silicon wafer that had a relatively high density of monolayer WSe_2 grown on it. This examination spotlighted the predominant diffraction peak at 13.62°, which aligns meticulously with the ICDD benchmark for the (002) facet of WSe_2. Moreover, an eminent peak observed at 33.02° prompted further inquiry. Subsequent XRD scrutiny of a pristine 285 nm SiO_2/Si substrate, devoid of WSe_2 growth, attributed such additional peaks, including the one at 33.02°, to the intrinsic diffraction signatures of the substrate. This observation is predominantly ascribed to firstly, the incomplete coverage of WSe_2 across the

SiO_2/Si substrate; and secondly, the inherently weak XRD signal emanation from the monolayer WSe_2, constrained by its dimensional and thickness limitations. Consequently, the XRD spectrum for the WSe_2 specimen predominantly features a pronounced peak near 13.62° corresponding to the (002) facet, whereas peaks associated with (006) and (008) facets manifest with diminished intensity. This concurs with the XRD outcomes for monolayer WSe_2 delineated in prior research[12-13].

References

1. Lee Y.H., Zhang X.Q., Zhang W.J.; Chang, M.T.; Lin, C.T.; Chang, K.D.; Yu, Y.C.; Wang, J.T.W.; Chang, C.S.; Li, L.J. et al. Synthesis of Large-Area MoS_2 Atomic Layers with Chemical Vapor Deposition[J]. Advanced Materials, 2012, 24(17): 2320-2325.
2. Sheng, Y.W.; Tan, H.J.; Wang, X.C.; Warner, J.H. Hydrogen Addition for Centimeter-Sized Monolayer Tungsten Disulfide Continuous Films by Ambient Pressure Chemical Vapor Deposition. Chem. Mater. 2017, 29, 4904−4911, doi:10.1021/acs.chemmater.7b00954.
3. Shaw, J.C., Zhou, H., Chen, Y. et al. Chemical vapor deposition growth of monolayer $MoSe_2$ nanosheets[J]. Nano Res., 2014, 7, 511–517.
4. Wang, X.L.; Gong, Y.J.; Shi, G.; Chow, W. L.; Keyshar, K.; Ye, G.; Vajtai, R.; Lou, J.; Liu, Z.; Ringe, E. et al. Chemical Vapor Deposition Growth of Crystalline Monolayer $MoSe_2$. ACS Nano 2014, 8, 5125-5131.
5. Zafar, A.; Zafar, Z.; Zhao, W.W.; Jiang, J.; Zhang, Y.; Chen, Y.F.; Lu, J.P.; Ni, Z.H. Sulfur-Mastery: Precise Synthesis of 2D Transition Metal Dichalcogenides. Adv. Funct. Mater. 2019, 29, 1-8, doi:10.1002/adfm.201809261.
6. Huang, J.K.; Pu, j.; Hsu, C.L.; Chiu, M.H.; Juang, Z.Y.; Chang, Y.H.; Chang, W.H.; Iwasa, Y.; Takenobu, T.S.; Li, L.J. Large-Area Synthesis of Highly Crystalline WSe_2 Monolayers and Device Applications. ACS Nano 2014, 8, 923-930, doi: 10.1021/nn405719x.
7. Liu, B.L. Fathi, M.; Chen, L.; Abbas, A.; Ma, Y.Q.; Zhou C.W. Chemical Vapor Deposition Growth of Monolayer WSe_2 with Tunable Device Characteristics and Growth Mechanism Study. ACS Nano 2015, 9, 6119–6127, doi:10.1021/acsnano.5b01301.
8. Alahmadi M.; Mahvash F.; Szkopek T.; Siaj M. A two-step chemical vapor deposition process for the growth of continuous vertical heterostructure WSe_2/h-BN and its optical properties[J]. RSC Adv, 2021, 11(28): 16962-16969.
9. Qiao, P.; Xia, J.; Li, X.; Li, Y.; Cao, J.; Zhang, Z.; Lu, H.; Meng, Q.; Li, J.; Meng, X.M. Epitaxial van der Waals contacts of 2D $TaSe_2$-WSe_2 metal-semiconductor heterostructures[J]. Nanoscale, 2023, 15(42): 17036-17044.
10. Wang, X.; Li, Y.; Zhuo, L.; Zheng, J.; Peng, X.; Jiao, Z.; Xiong, X.; Han, J.; Xiao, W. Controllable growth of two-dimensional WSe_2 using salt as co-solvent[J]. CrystEngComm., 2018, 20(40): 6267-6272.
11. McCreary, K.M.; Hanbicki, A.T.; Jernigan, G.G.; Culbertson J.C.; Jonker, B.T. Synthesis of Large-Area WS_2 monolayers with Exceptional Photoluminescence. Sci. Rep. 2016, 6, 1-7, doi:10.1038/srep19159.

12. Kim, H.; Yun, S.J.; Park, J.C.; Park, M.H.; Park, J.H.; Kim, K.K.; Lee, Y.H. Seed Growth of Tungsten Diselenide Nanotubes from Tungsten Oxides. Small 2015, 11, 2192–2199, doi:10.1002/smll.201403279.
13. Zhang, Y.X.; Wang, Y.H.; Xiong, Z.Z.; Zhang, H.J.; Liang, F. Preparation and characterization of WSe2 nano-films by magnetron sputtering and vacuum selenization. Nanotechnology 2018, 29, 1-7, doi: 10.1088/1361-6528/aabab0.
